# Gaussian curvature dilutes the nuclear lamina, favoring nuclear rupture, especially at high strain rate

**DOI:** 10.1080/19491034.2022.2045726

**Published:** 2022-03-16

**Authors:** Charlotte R. Pfeifer, Michael P. Tobin, Sangkyun Cho, Manasvita Vashisth, Lawrence J. Dooling, Lizeth Lopez Vazquez, Emma G. Ricci-De Lucca, Keiann T. Simon, Dennis E. Discher

**Affiliations:** a Physical Sciences Oncology Center at Penn (PSOC@penn), University of Pennsylvania, Philadelphia, PA, USA; b Molecular & Cell Biophysics Lab, University of Pennsylvania, Philadelphia, PA, USA; c Graduate Group/Department of Physics & Astronomy, University of Pennsylvania, Philadelphia, PA, USA; d Graduate Group/Department of Bioengineering, University of Pennsylvania, Philadelphia, PA, USA

**Keywords:** curvature, nuclear lamina, nuclear envelope rupture, myosin stress, biophysical model, mechanobiology

## Abstract

Nuclear rupture has long been associated with deficits or defects in lamins, with recent results also indicating a role for actomyosin stress, but key physical determinants of rupture remain unclear. Here, lamin-B filaments stably interact with the nuclear membrane at sites of low Gaussian curvature yet dilute at high curvature to favor rupture, whereas lamin-A depletion requires high strain-rates. Live-cell imaging of lamin-B1 gene-edited cancer cells is complemented by fixed-cell imaging of rupture in: iPS-derived progeria patients cells, cells within beating chick embryo hearts, and cancer cells with multi-site rupture after migration through small pores. Data fit a model of stiff filaments that detach from a curved surface.Rupture is modestly suppressed by inhibiting myosin-II and by hypotonic stress, which slow the strain-rates. Lamin-A dilution and rupture probability indeed increase above a threshold rate of nuclear pulling. Curvature-sensing mechanisms of proteins at plasma membranes, including Piezo1, might thus apply at nuclear membranes.

**Summary statement:** High nuclear curvature drives lamina dilution and nuclear envelope rupture even when myosin stress is inhibited. Stiff filaments generally dilute from sites of high Gaussian curvature, providing mathematical fits of experiments.

## Introduction

**Figure 1.**
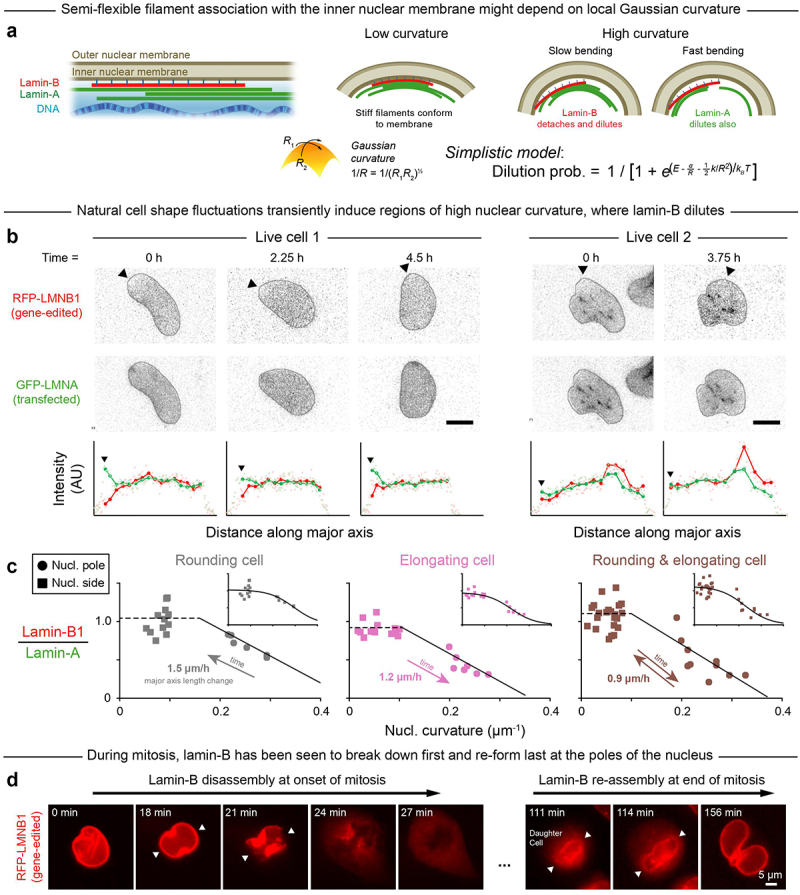
High Gaussian curvature disrupts the nuclear lamina.

During interphase, the nuclear envelope typically functions as a barrier between the nucleus and cytoplasm. However, rupture of the envelope mis-localizes key nuclear proteins to cytoplasm that include transcription factors [1] and DNA repair factors [2,3], while anomalous nuclear entry includes some DNA-binding proteins [3–6]. Rupture mechanisms continue to be elucidated, especially biophysical determinants. Early work associated rupture with deficiencies or defects in lamins [7–10]. A- and B-type lamins make ~^1^/_2_ µm-long filaments that resist bending within juxtaposed meshworks at the inner nuclear membrane [11], and farnesylated lamin-B1 and -B2 associate more directly with the membrane than non-farneslyated lamin-A,C [12,13] (Figure 1a). Lamin-B levels are nearly constant across different tissues [14] and double in level as a cell duplicates its DNA [15], whereas lamin-A,C increases from low levels in soft embryos and brain to high levels in stiff muscle and rigid bone [14,16]. Various cells studied here are representative with lamin-A:B ratios that range from ~1:1 in early chick embryo hearts [16] to ~2:1 in A549 human lung cancer cells [17] and ~7:1 in mesenchymal stem/progenitor cells derived from progeria patient iPS cells [18]. The diversity of cell types studied here helps to provide general insight into how lamins confer nuclear strength and stability.

A. A-type and B-type lamins form juxtaposed networks inside the nuclear membrane. Lamin filaments are expected to stably interact with a membrane of low Gaussian curvature but to dissociate from a membrane with high Gaussian curvature (see ‘Single-filament model,’ Materials & Methods). *R* = radius of Gaussian curvature; *R*_1_,*R*_2_ = principal curvature radii. Lamin-A and lamin-B networks are thought to have differing material properties – and hence to dilute, or be depleted, at differing rates from regions of high nuclear curvature. B. Low-light imaging: A549 human lung cancer cells with RFP-lamin-B1 from monoallelic gene editing were transfected with GFP-lamin-A and live-imaged periodically over hours. Cell shape fluctuations transiently induced regions of high nuclear curvature. Intensity profiles: measured along the cell’s major axis, originating at the arrow head. Representative of 10 cells/20 nuclear poles; scale = 10 µm. C. Lamin-A and -B intensities were measured at the poles and sides of nuclei in three representative cells that rounded and/or elongated during live-imaging. Plots show lamin-B to lamin-A intensity ratio as a function of nuclear curvature. Insets: same data, fit with a sigmoidal function per our simple filament (un)binding model. Representative of 4 cells/8 nuclear poles. D. Live images of A549 mitosis, with the lamin-B network disassembling first and reassembling last at the higher-curvature poles of the nucleus (arrow heads).

Tension in the nuclear membrane might cause rupture and could result from pressure differences among other forces [19]. Continuity of the outer nuclear membrane with the endoplasmic reticulum would tend to dissipate high tensions and oppose nuclear membrane rupture, which suggests additional mechanisms. High-curvature AFM tips cause nuclear-specific rupture when pressed on nuclei within intact adherent cells [20], and high-curvature micronuclei are also most susceptible to rupture [21]. Although it has been proposed that lamins-A and -C but not lamin-B1 regulate nuclear mechanics [22], the meshwork of lamin-B is frequently disrupted at nuclear poles (distinctly from lamin-A,C) after migration through small pores [17] or narrow channels [4, 6]. Some of these observations began to associate nuclear envelope rupture with positive Gaussian curvature [20, 21] as opposed to simple mean curvature (Figure S1A). Relationships otherwise remain unclear between site(s) of nuclear rupture and nuclear curvature, including negative Gaussian curvature (saddle shapes), and time- or rate-dependent density changes of A- and B-lamins.

Based on the known correlation between nuclear rupture and lamin deficiency, we hypothesized that rupture associates with regions of high positive Gaussian curvature because such curvature locally dilutes, or depletes, the nuclear lamina. Distinctive effects might be expected for the lamin-A and lamin-B networks because they, respectively, exhibit viscoplastic or elastic behavior [14]. Thus, we further hypothesized that the elastic lamin-B meshwork would dilute comparatively quickly in regions of high curvature due to weakened interactions and rapid detachment of stiff lamin-B filaments from the highly curved nuclear membrane. By contrast, the local density of the more viscoplastic lamin-A would depend more strongly in its response on the rate of change of nuclear curvature (Figure 1a). We envisioned that the curvature sensitivities of lamin-A and lamin-B bear some relation to those of the membrane mechanosensors Piezo1 and Piezo2. These large channel proteins exhibit activity that is sigmoidal in its fitted stress-dependence [23] and is modeled as depending on membrane curvature with modulation by cytoskeleton [25]. Discovery of Piezo’s mechanosensitivity [24] was the subject of the 2021 Nobel Prize in Physiology or Medicine, and the evident importance motivates deeper understanding of any curvature-dependent response of the nuclear lamina.

## Results and Discussion

### Dilution of lamin-B at nuclear poles fits a simple equilibrium model

We first imaged local changes in lamin levels as nuclear curvature fluctuated over hours within sparsely plated A549 cells that spread, elongated, and/or rounded-up in standard culture. The cells were gene-edited to express RFP-lamin-B1 (‘Cancer cell lines,’ Materials & Methods) in order to minimize strong effects of lamin-B1 perturbations on cell cycle [10,25], and they were also transfected with GFP-lamin-A for live-imaging. Lamin-B1 was often diluted in regions of high curvature unlike lamin-A (Figure 1b). Regardless of the quasi-static nuclear extension or rounding, the lamin B-to-A intensity ratio was low at the nuclear poles but not the flat sides or other low-curvature regions (Figure 1c). Immunostaining after fixation of distinct A549 clones with persistent ‘spindle-shaped’ nuclei confirmed the relative dilution of lamin-B at high-curvature nuclear poles, as did imaging of U2OS human bone cancer cells (Figure S1B-D); these analyses of immunostained cells showed that such dilution occurs with or without the presence of fluorescent protein tags.

The live-imaging results for curvature-dependent lamin-B dilution fit a parsimonious, ‘spherical cow’-type ‘Single-filament model’ (Figure 1a;Materials & Methods). In the model, semi-flexible lamin-B filaments stably bind (energy *E*) to an inner nuclear membrane of low Gaussian curvature but incur increasing binding energy costs (~1/*R*) and bending energy costs (~1/*R*^2^) as membrane curvature increases. As a result, the probability of lamin-B filament detachment also increases (sigmoidally) with membrane curvature, which fits the live-imaging data. Lamin filaments have a median persistence length of ~^1^/_2_ µm and can have persistence lengths of up to ~3 µm [11], which means that some filaments are indeed rigid on the ~micron-length scale of membrane curvature at nuclear poles. While additional mechanisms might contribute to lamin-B dilution at high curvature, model agreement with the initial live-imaging results starts to suggest that lamin-B dilution is caused by filament detachment from highly curved membranes. Meanwhile, lamin-A, which is not farnesylated and can be nucleoplasmic especially when phosphorylated [14,16,18], associates more weakly with the inner nuclear membrane [12,13], and it is seen to dilute minimally at the low deformation rates typical of cell and nuclear movements in 2D culture.

Mitotic cells further show the lamin-B meshwork disassembled first at the highest curvature nuclear poles and also re-assembled last at such sites (Figure 1d, Figure S2). Nuclear curvature might thus contribute to lamin-B disassembly-reassembly in mitosis together with other biophysical and established molecular mechanisms [26,27]. Whereas holes in the lamin-B network are typical of the mitotic initiation of nuclear envelope breakdown [28,29], local depletion of lamin-B in interphase nuclei can lead to aberrant nuclear rupture.

### Rupture at nuclear poles in progeria-derived cells and within chick embryos

**Figure 2.**
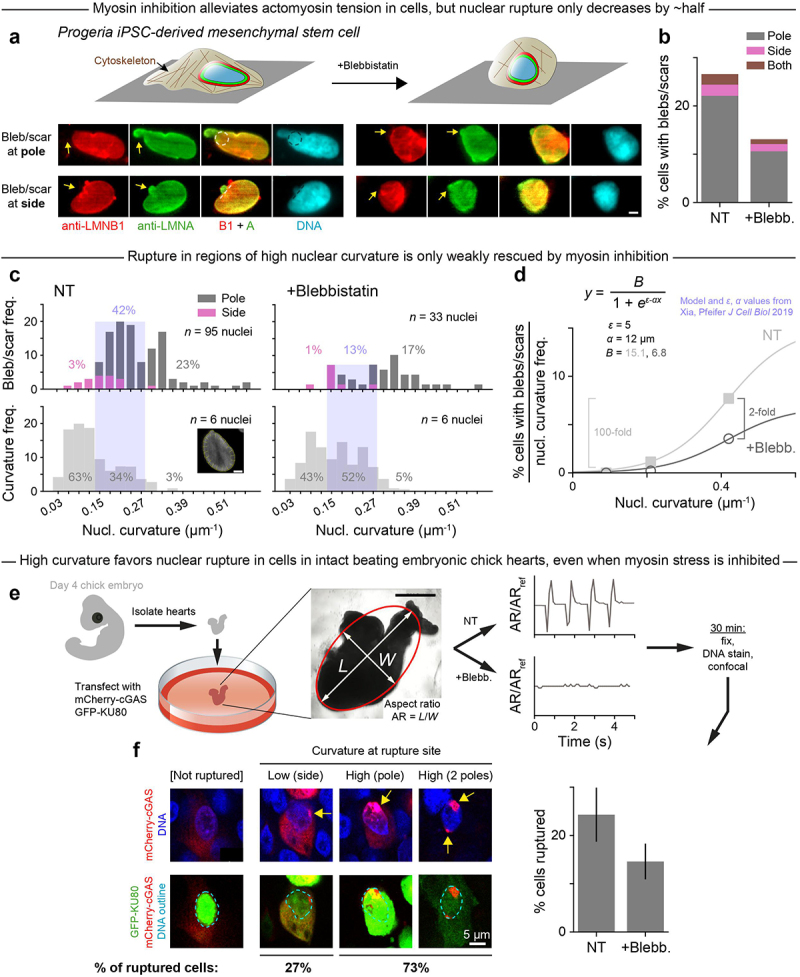
In progeria cells and cells in intact beating embryonic chick hearts, high Gaussian curvature favors nuclear rupture, even when myosin stress is inhibited.

To further investigate any relationship between curvature, lamin-B depletion, and aberrant nuclear rupture in interphase nuclei, mesenchymal stem/progenitor cells were differentiated from iPS cells with some mutant lamin-A from patients with Hutchinson-Gilford progeria syndrome [18,30,31]. Progeria cells were chosen as a model system because their defective, farnesylated lamin-A should favor nuclear rupture in cultures on rigid substrates that promote actomyosin stress in the spreading of the cell and its nucleus [8,20]. Frequent rupture was indeed evident as lamin-B-deficient blebs (Figure 2a). Importantly, the blebs that contain normal lamin-A and perhaps mutant lamin-A again occurred overwhelmingly at the poles of the nucleus (Figure 2b), confirming that high curvature correlates not only with lamin-B dilution but further with nuclear envelope rupture.

A. Mesenchymal stem/progenitor cells from patients with progeria have a defective nuclear lamina, which increases nuclear rupture in standard 2D culture. The nuclear bleb or scar shows abundant lamin-A but loss of lamin-B (yellow arrows). Myosin-II inhibitor blebbistatin reduces stress on the nucleus, but blebs/scars persist. iPSC = induced pluripotent stem cell. Images: dashed circles and lines trace the local curvature; scale = 5 µm. B. Rupture frequency, as indicated by % progeria cells with blebs/scars, is highest at the nuclear pole and decreases by ~half after blebbistatin (blebb.) treatment. NT = non-treated; 594 cells; ≥ 242 cells per condition. C. Lower: For 6 non-treated and 6 blebb.-treated progeria cells (randomly selected), curvature was measured at ~20 locations around the nuclear perimeter (inset image, scale = 5 *µ*m). Plots show the curvature distributions. Upper: For progeria cells from panel B with blebs/scars, nuclear curvature at the location of the bleb/scar was measured, with blue shading distinguishing low, medium, or high curvature. The latter two are enriched for blebs/scars. D. For each of the three nuclear curvature regimes from panel C (low, medium, high), the frequency at which a rupture-inducing curvature falls in that regime (panel C upper) was normalized to the frequency at which that regime occurs in progeria cell nuclei (panel C lower) – and then plotted against the median curvature for that regime. Sigmoidal fit parameters come from our simple filament (un)binding model. E. Hearts isolated from chick embryos were transfected with the nuclear protein GFP-KU80 and the cytoplasmic DNA-binding protein mCherry-cGAS, and then treated with blebb. or control DMSO. During the 30-min live-imaging that followed, blebb. suppressed beating of the heart aspect ratio AR_ref_. *L, W* = length, width. Representative of six hearts; scale = 200 µm. F. Isolated hearts from panel E were fixed, DNA stained, and imaged. Some heart cells showed nuclear envelope rupture (bar plot) based on nuclear accumulation of cGAS (upper images: yellow arrows) and mis-localization of KU80 outside the nucleus (lower images: dashed outlines indicate nucleus). Seventy-two cells; ≥18 cells per condition; mean ± SEM.

It remained unclear whether rupture is driven by curvature itself or by actomyosin-generated stresses that compress the nucleus into an elongated shape with high-curvature regions. To address this question, we inhibited myosin II and observed rounding of the cells and their nuclei (Figure 2a), yet rupture frequency decreased by only ~half overall (Figure 2b). The rescue effect was even weaker at high curvature: from the distributions of all ruptures observed in non-treated and blebbistatin (blebb.)-treated progeria cell nuclei, we calculated the percentage of ruptures that occurred at low, medium, and high curvature (Figure 2c). We found that the share of low- and medium-curvature ruptures fell by ~two-thirds after blebb. treatment; meanwhile, the share of ruptures that occurred at high curvature was more similar in non-treated and blebb.-treated nuclei. These results imply that high curvature-associated rupture is largely independent of myosin stress and is instead driven by curvature itself. High curvature-associated rupture also accounts for a disproportionately large percentage of all ruptures given the relative rarity of high-curvature regions in progeria cell nuclei (Figure 2c, lower). We normalized the percentage of ruptures in the low, medium, and high curvature regimes to the frequency at which those curvature regimes occur (Figure 2d). This analysis highlighted that, in contrast to the relatively modest two-fold overall decrease with myosin inhibition, rupture was virtually eliminated in the large (frequent) regions of low nuclear curvature in both non-treated and blebbistatin-treated cells. This strong dependence of rupture on curvature again fits our simple model of a semi-flexible lamin filament that binds or not to a highly curved nuclear surface (Figure 2d), which reinforces the idea that curvature-induced lamin-B loss precipitates nuclear envelope rupture.

To address whether curvature-associated rupture also occurs in intact tissues, beating hearts were isolated from day-4 chick embryos, transfected with two factors that reveal nuclear rupture [16], treated or not with blebbistatin, and then fixed, DNA-stained, and imaged by confocal microscopy (Figure 2e). Rupture was assessed by the cytoplasmic presence of the DNA repair factor GFP-KU80 in the same cells that also show focal nuclear entry of DNA-binding protein mCherry-cGAS. Myosin inhibition stopped the beating but again caused only a modest decrease in nuclear rupture, with the rupture occurring mostly at the nuclear poles (Figure 2f). Intact tissue results thus point once again to high curvature, more so than myosin stress, as a predominant driver of nuclear rupture.

### Multiple ruptures in the same nucleus conforms to independent probabilities

Some nuclei in the hearts showed multiple sites of rupture (Figure 2f) as was also the case for the progeria cells, suggesting that rupture can occur in a nucleus with a preexisting bleb. Phospholipid giant vesicles studied in viscous media by Brochard-Wyart and colleagues [32,33] likewise showed multiple sites of rupture. Each rupture resolved before the next, indicating that high internal pressure caused a vesicle to develop a hole and leak fluid, alleviating pressure, allowing the hole to reseal, and repeating with dozens of transient holes in succession. Even without rupture, plasma membrane bleb formation in fibroblasts has been shown to significantly relax intracellular pressure that returns to its initial high value only after the bleb retracts [34]. If nuclear rupture is thus primarily driven by intranuclear pressure that is reduced upon either rupture or blebbing, then rupture events are *not* independent (of one another or of preexisting pressure-alleviating blebs). If, however, curvature effects supersede pressure modulation in nuclear envelope rupture, then the persistence of one bleb should be independent of further ruptures (and accompanying blebs) at additional high-curvature sites. Regardless of pressure effects or not in nuclear rupture, we wondered whether our single-filament model might also describe multi-site rupture.

**Figure 3.**
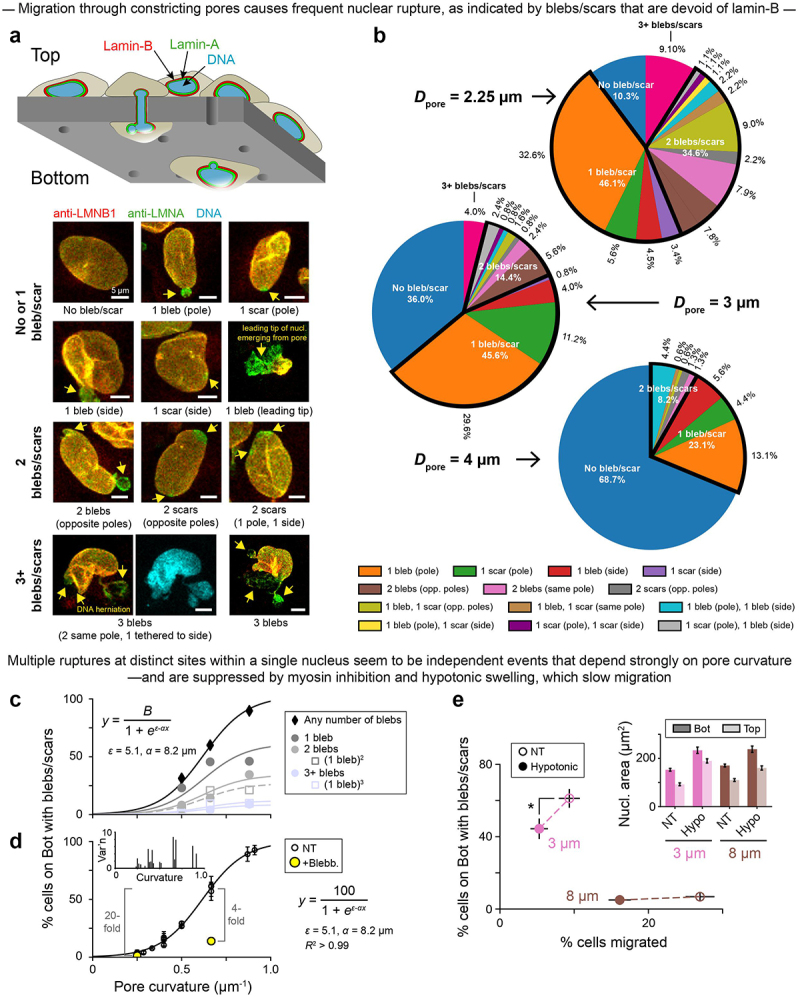
Migration through constricting pores causes frequent nuclear rupture; multiple such ruptures at distinct sites within a single nucleus seem to be independent events that depend strongly on pore curvature.

To assess the independence of multi-site nuclear rupture, painstaking statistics of the number of lamin-B deficient blebs or scars on U2OS nuclei were made after cell migration through constricting pores of different diameters, either custom-made or commercially available (Figure 3a,b). Such migration has been seen to squeeze out nucleoplasmic proteins [2], which implies a buildup of nuclear pressure. A single rupture again follows the simple sigmoidal probability *P*_rupt_ as a function of pore-imposed curvature, as is expected if the probability of rupture *P*_rupt_ is linearly proportional to, and thus has the same functional form as, the probability of lamin-B dilution (Figure 1a, Eqn). More importantly, the fraction of nuclei with either two ruptures or three (or more) fit to (*P*_rupt_)^2^ or (*P*_rupt_)^3^, respectively (Figure 3c). These findings imply independent events and argue that high Gaussian curvature drives rupture with or without excess intranuclear pressure. Although curvature and pressure can be complementary drivers of nuclear rupture, the analyses suggest that curvature is sufficient to explain the observations. Nuclear pressure changes during rupture events must therefore be measured directly rather than presumed or inferred from volume changes.

A. Nuclei rupture as cells migrate through constricting pores from the Top to the Bottom of a Transwell. Images: nuclear blebs and scars (yellow arrows) in migrated U2OS human bone cancer cells on the Bottom of a 3 µm pore membrane. Some nuclei show multi-site rupture, with 2 or 3+ blebs/scars. B. Distributions of bleb/scar number and location among U2OS cells that have migrated through pores of varying diameter *D*_pore_. ≥ 90 cells per *D*_pore_. C. Rupture frequency during migration, as indicated by % migrated U2OS cells with blebs/scars, increases with pore curvature. This trend holds overall (black diamonds) as well as among migrated cells with 1 (dark gray), 2 (light gray), and 3+ (light blue) rupture sites. Filled points are measured values from panel B. Unfilled are estimates of multi-site rupture frequency, obtained by treating each rupture as an independent event with probability equal to the measured % of 1-bleb/scar cells. Sigmoidal fits are per our simple filament (un)binding model; from top to bottom curve, fit parameter *B* = 102, 61, 34, 27, 12, 9. D. Across an expanded range of pore diameters (*D*_pore_ = 3 µm to 8 µm), rupture frequency depends strongly on pore curvature. Addition of blebb. to the Bottom of the pore membrane suppresses rupture but not as much as a ~2.5-fold curvature reduction. Data adapted from Xia, Pfeifer 2019; ≥ 50 U2OS cells per *D*_pore_; mean ± SEM. Inset: SEM values, from the main plot, are highest at intermediate pore curvatures, consistent with the derivative of the main curve. E. Hypotonic medium was applied to the Top and Bottom of 3 and 8 µm pore membranes during U2OS cell migration. Non-treated (NT) = isotonic medium. Three hundred and three cells. Inset: hypotonic medium increases nuclear area across all conditions. Five hundred and thirty-nine cells; ≥ 39 cells per condition; mean ± SEM.

Actomyosin inhibition – which should suppress nuclear pressure – is again less effective than low curvature in rescuing rupture (Figure 3d): the former gives a 4-fold decrease in rupture frequency versus a 20-fold decrease for the latter. Likewise, even when migrating cells were subjected to hypoosmotic stress, which caused nuclear swelling consistent with higher intranuclear pressure (Figure 3e**, inset**), rupture frequency *decreased* (Figure 3e, Figure S3A), as did average bleb size (Figure S3B). Multi-site rupture again suggested independent events (Figure S3C). Importantly, both hypoosmotic stress (Figure 3e) and actomyosin inhibition [21] cause slower migration to the bottom of the Transwell device. This finding motivated a final, controlled study of nuclear strain rate.

### Lamin-A dilution is delayed, but low lamin-A maximizes curvature-driven rupture

**Figure 4.**
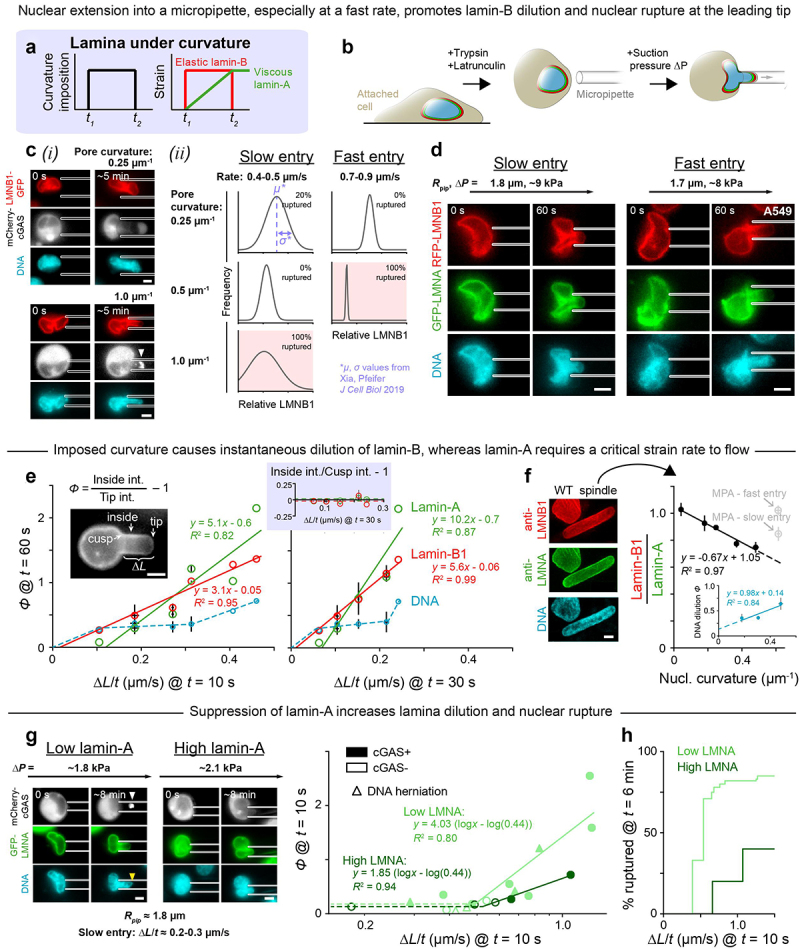
Nuclear entry into a constriction causes instantaneous dilution of lamin-B at the leading tip of the nucleus, while lamin-A requires a critical strain rate to flow.

Slow curvature changes seem to result in little to no dilution of the supposedly viscous lamin-A meshwork (Figure 1a-c, Figure S1B,C; Figure 4a). To further investigate the effects of strain rate on lamina dilution and nuclear rupture, U2OS cells expressing lamin-B1-GFP were detached, actin-depolymerized and pulled into micropipettes of varying diameter at either a slow or fast rate (Figure 4b,c). Regardless of the aspiration rate, nuclei always ruptured in the highest-curvaturesmall-diameter pipettes and rarely ruptured in the lowest-curvature pipettes (Figure 4c-ii), consistent with results for constricting pores (Figure 3c,d). This experiment also revealed an association between rapid curvature imposition (i.e. fast nuclear extension into the pipette), lamin-B dilution at the leading tip to some extent, and rupture (Figure 4c-ii), which lends credence to the observation that slow migration under hypoosmotic stress could suppress rupture.

A. Lamin-A and lamin-B networks are predicted to have viscous and elastic responses, respectively, to applied stress. B. Cells were detached, treated with latrunculin to depolymerize the actin cytoskeleton and pulled under controlled pressure ∆*P* into micropipettes. C. U2OS cells were transfected with lamin-B-GFP and mCherry-cGAS. (*i*) Representative images of cell aspiration into a low-curvature or a high-curvature pipette. Arrow head = nuclear envelope rupture, indicated by nuclear accumulation of cGAS. Scale = 5 µm. (*ii*) For 17 cells pulled at a slow or fast rate into pipettes of varying curvature, lamin-B intensity at the nuclear tip was measured at *t* = 15 s. Plots show distributions of these intensities for all conditions. Based on mean *µ*± SEM σ values from Xia, Pfeifer 2019; ≥ 3 cells per condition. D. A549 cells with gene-edited RFP-lamin-B were transfected with GFP-lamin-A and aspirated at varying rates into pipettes of fixed curvature ≈ 0.5 µm^−1^ to examine resulting lamina dilution. *R*_pip_ = pipette radius. E. For 14 aspirated cells (panel D), nuclear tip dilution *ϕ* of lamin-A, lamin-B, and DNA was calculated (left inset) at *t* = 60 s. Plots show *ϕ* as a function of nuclear entry rate into the pipette Δ*L*/*t*, measured at an early timepoint (left) and slightly later timepoint (right). Right inset shows that, regardless of nuclear entry rate, lamins-A and -B do not dilute at the cusp at the pipette tip. One to four cells per bin; mean ± SEM for each bin. F. Images: select ‘spindle-shaped’ A549 clones have stably elongated nuclei as compared to wild-type (WT). Scale = 5 µm. Plot: measurements taken at poles of spindle-shaped nuclei in 2D culture (black points) and at the leading tip of WT nuclei in micropipettes (gray points, data from panel E). Two hundred and ninety-eight spindle-shaped cells; ≥ 11 cells per bin; mean ± SEM for each bin. Inset plot: In the same spindle-shaped nuclei, dilution *ϕ* of DNA was calculated at the nuclear poles as *ϕ=* (center int.)/(pole int.) – 1, where int. = intensity. Like lamin-B, DNA dilutes at regions of high nuclear curvature. One hundred and fifty poles; mean ± SEM. G. U2OS cells with lamin-A knockdown were transfected with GFP-lamin-A – yielding a wide range of lamin-A levels – and mCherry-cGAS. Images: aspiration of a low-lamin-A and a high-lamin-A cell into a pipette of curvature ≈ 0.5 µm^−1^. Arrow heads = nuclear envelope rupture, indicated by nuclear accumulation of cGAS (white) and DNA herniation (yellow). Scale = 5 µm. Plot: nuclear tip dilution *ϕ* of lamin-A versus nuclear entry rate into the pipette Δ*L*/*t*. Solid lines are fitted to the 11 low-lamin-A and 4 high-lamin-A cells with the highest Δ*L*/*t*; dashed lines guide the eye. cGAS± = does/does not exhibit nuclear cGAS accumulation. H. Low lamin-A cells rupture with enhanced frequency at a slow nuclear extension rate. Cells from panel G. % ruptured comprises cGAS+ cells and/or cells with DNA herniation.

The micropipette aspiration experiment was repeated with the endogenously tagged RFP-lamin-B1 A549 cells transfected with GFP-lamin-A. Past aspiration studies of the latter had been combined with photobleaching to show some stretching and dilution of the lamin-A meshwork on the cylindrical projection, but rate effects and rupture were not examined. The cells here were pulled at controlled rates into pipettes of curvature ~0.5 µm^−1^ (Figure 4d,e, Figure S4A). As a nucleus extended into the pipette, lamin-B diluted instantaneously at the leading tip, proportional to extension rate ∆*L*/*t*, whereas lamin-A diluted only above a critical extension rate (∆*L*/*t*)_crit_ at the same timepoint in aspiration (Figure 4e). At very long times, lamin-A diluted more like lamin-B and independent of initial rate (Figure S4B). Notably, neither lamin-A nor lamin-B diluted at the cusp created by the pipette wall (Figure 4e), which suggests that both principal curvatures at a site must be the same sign (Figure S1) – as at a nuclear pole – to cause lamin filament dissociation and dilution at that site. Dilution of DNA seemed similar to that of lamin-B, occurring even at slow extension rates below (∆*L*/*t*)_crit_ (Figure 4e).

Because extension rates below (∆*L*/*t*)_crit_ were difficult to probe with aspiration, we studied the ‘spindle-shaped’ A549 clones that exhibit a micropipette-like curvature but under slow, cell-generated forces in 2D culture (Figure S1B,C): lamin-B dilutes at the highly curved nuclear poles (Figure 4f), as does DNA (Figure 4f, inset). Co-dilution of lamin-B and DNA at sites of high nuclear curvature points to possible involvement of chromatin in lamina disruption and nuclear rupture, though much more study is needed. Meanwhile, lamin-A does not dilute at the highly curved nuclear poles of ‘spindle-shaped’ cells (Figure 4f). The lamin-A layer thus requires a high strain rate to flow and be depleted. This rate-dependent behavior is typical of viscous materials and might underlie the association of slow migration and reduced nuclear rupture in both hypotonically swollen and actomyosin-inhibited cells. Thus, when curvature imposition is slow, lamin-A dilutes minimally or not at all, allowing it to fortify the nuclear envelope against rupture even with lamin-B dilution. Consistent with such a mechanoprotective role for lamin-A, low-lamin-A cells exhibit greater lamina dilution and more frequent nuclear rupture when compared to high-lamin-A cells (Figure 4g,h, Figure S4C-E).

Overall, our results support a model in which lamin-B filaments stably bind to low-curvature nuclear membranes but are too stiff to bend along high-curvature membranes – and thus detach quickly when curvature is applied. Alternative and complementary mechanisms of lamin-B dilution remain possible: for example, high curvature could cause the lamin-B meshwork to stretch and distend, increasing the distance between filaments and reducing their density locally. Distension could be accompanied by other forms of lamin-B reorganization, including sliding of lamin-B filaments along the inner nuclear membrane [35], or could even precipitate structural changes in the lamin-A layer that are known to be mechanically induced [36,37]. These alternative mechanisms warrant further study. However, data from multiple cell types, across a variety of assays, consistently fit a parsimonious model of a stiff filament that detaches from a curved surface (Figure 1a, Figure 2d, Figure 3c,d), providing initial evidence that lamin filament detachment underlies lamina dilution and ultimately nuclear envelope rupture.

Interestingly, chromatin seems to instantaneously dilute alongside lamin-B at regions of high nuclear curvature (Figure 4e,f), suggesting a possible role for chromatin in lamina disruption and nuclear rupture. The causes and consequences of chromatin dilution remain to be determined; studies performed under conditions of variable chromatin condensation, as in [38], would be informative. In comparison to lamin-B and DNA, lamin-A requires a critical strain rate to flow: for slow curvature imposition, the lamin-A layer remains minimally deformed, but for rapid curvature imposition, lamin-A dilutes with lamin-B, and the envelope ruptures with high frequency. Interestingly, the method, which shows that local, transient tensile stress on the nuclear membrane causes membrane rupture [19] also causes high nuclear curvature at the rupture site. Likewise, in this study, across multiple primary cell types and established lines, and with multiple experimental strategies, actomyosin stress and intranuclear pressure are relatively weak modulators of lamina dilution and nuclear rupture compared to strong effects of positive Gaussian curvature. Thus, while actomyosin tension, intranuclear pressure, or possibly other forces can contribute to nuclear envelope rupture, the data presented here argue that Gaussian curvature is the predominant driver of such rupture.

Lamin deficient or defective mice exhibit nearly all of the basic tissue lineages, which indicates minimal defects in differentiation, and although they are near-normal in size up to birth, they exhibit some key tissue-specific defects in proliferation. Notably, lamin-B mutants show rupture and proliferation defects in brain [10]. Because lamin-A is very low in soft brain, brain cells normally depend on lamin-B. In contrast, stiffer tissues have high lamin-A and will be protected from lamin-B defects, and the interested reader is encouraged to review the extensive literature on lamin-A mutants with such a perspective. Deeper physical insight into interphase rupture mechanisms thus motivates a better understanding of lamin stoichiometry and levels per DNA in addition to lamin interactions (farnesylation, lamin-B receptor, etc.). Understanding the basics of rupture as a biophysical process is also foundational to clarifying its effects on DNA damage, cell cycle disruption, senescence, and premature death of lamin defective organisms, from mouse to human.

## Materials and methods

*Single-filament model* As previously described [21] and in the spirit of a mean-field model, we consider a single lamin-B filament that is stiff with an *in situ* length *L*_fil_ and persistence length ℓ_p_ [11]. The filament is either attached to or detached from the nuclear membrane; hence, in this parsimonious model, we write the following partition function for the filament:

(1) $$Z=\;\sum_{s}e^{-E_{s}/k_{B}T}=e^{-E_{attached}/k_{B}T}+e^{-E_{detached}/k_{B}T}$$

We take the detached state to be the reference state with *E*_detached_ = 0. If the nuclear membrane were flat, *E*_attached_ would simply be equal to a negative binding energy, -*E*, that favors filament attachment. But since the nuclear membrane can be curved (with curvature = 1/*R*), we consider two contributions to the energy of the attached state:

(2) $$E_{attached}=E_{binding}+E_{bending}$$

*E*_bending_ is the energy cost for bending the stiff filament along the curved membrane, and it is given by $\frac{1}{2}\frac{k}{R^{2}}$, where *k* is the filament bending constant. *E*_binding_ is not simply equal to -*E* but is instead modulated by membrane curvature since curvature changes the filament-membrane contact area. Altogether, we can rewrite Eq. 2 as a function of curvature:

(3) $$E_{attached}=-E+\frac{a}{R}+\frac{1}{2}\frac{k}{R^{2}},$$

with *a*/*R*, where *a* is a constant, capturing the curvature-induced change in contact area between the nuclear membrane and the lamin-B filament. The second and third terms increase with 1/*R*, meaning that filament attachment becomes more energetically costly at high membrane curvatures – and detachment becomes more likely. The probability of the detached state is:

$$P_{detached}=\frac{1}{Z}e^{-E_{detached}/k_{B}T}$$

(4) $$P_{detached}=\frac{1}{1+e^{\left(E-\frac{a}{R}-\frac{1}{2}k/R^{2}\right)/k_{B}T}}$$

Indeed, in the high-curvature limit, 1/*R* becomes very large such that

$$e^{\left(E-\frac{a}{R}-\frac{1}{2}k/R^{2}\right)/k_{B}T}\ll 1$$

and *P*_detached_ → 1, consistent with a high probability of lamin-B dilution and nuclear envelope rupture at the high-curvature poles of nuclei (Figure 1c, Figure 2d) and in small pores (Figure 3c,d) and pipettes (Figure 4c). Meanwhile, in the low-curvature limit, 1/*R* → 0, *P*_detached_ → $1/\left(1+e^{E/k_{B}T}\right)\to$ 0, assuming *E* ≫ *k_B_T*. That is, a lamin-B filament is unlikely to detach from a nuclear membrane of low curvature, which is consistent with a low probability of lamin-B dilution and rupture on the flat sides of nuclei or in large pores and pipettes.

Note that the filament bending constant *k* depends on the bending stiffness of the filament, ℓ_p_*k*_B_*T*, and – because the bending energy is integrated over the whole length of the filament – the *in situ* length *L*_fil_: *k* = (ℓ_p_*k*_B_*T*)*L*_fil_. We use the values *L*_fil_ = 0.38 µm and ℓ_p_ = 0.5 µm from [11] to calculate *k* = (ℓ_p_*k_B_T*)*L*_fil_ = (0.5 µm)(0.38 µm)*k_B_T* = (0.19 µm^2^)*k_B_T*. When fitting data (Figure 2d, Figure 3c,d), we find that the bending term $\frac{1}{2}\frac{k}{R^{2}}$ has almost no effect on the best-fit curve, so we frequently exclude it. Fits are thus often of the form

(5) $$y=\frac{B}{1+e^{\varepsilon -\alpha x}},$$

where *B* is a fit parameter and *ε* and *α* are, respectively, a binding energy and an interaction energy that were previously determined [21].

*Cancer cell lines* U2OS human osteosarcoma cells were a gift from Dr. Roger A. Greenberg of the University of Pennsylvania, Philadelphia, USA. A549 human lung adenocarcinoma cells with endogenously RFP-tagged lamin-B1 were purchased from MilliporeSigma (product number: CLL1149). The *LMNB1* gene was tagged using CompoZr® zinc finger nuclease technology, resulting in a fusion protein in which RFP is attached to the N-terminus of LMNB1, as described in [39]. U2OS and A549 cells were cultured in DMEM high glucose medium (Gibco) and Ham’s F12 medium (Gibco), respectively, and both were supplemented with 10% fetal bovine serum (FBS; MilliporeSigma) and 1% penicillin/streptomycin (Gibco). Cells were kept at 37°C and 5% CO_2_.

*Progeria MSCs* Primary fibroblast-derived induced pluripotent stem cells (iPSCs) were acquired from the Progeria Research Foundation Cell & Tissue Bank. iPSC differentiation into mesenchymal stem cells (MSCs) was performed as described in [40]. Three days after initial splitting, iPS culture medium was replaced with MSC medium (low glucose DMEM supplemented with 10% FBS and 1% penicillin/streptomycin), which was then refreshed every 2 days. After 2 weeks of culture, cells were detached using 0.25% trypsin-EDTA (Gibco) and expanded on 0.1% gelatin coated dishes (BD Biosciences); cells were passaged whenever they reached confluence. After the third passage, a homogenous fibroblast-like cell population was achieved.

*Embryonic chick hearts* Embryonic chick hearts were extracted from White Leghorn eggs (Charles River Laboratories; Specific Pathogen Free (SPF) Fertilized eggs, Premium #10,100,326). SPF eggs were incubated at 37°C and 5% CO_2_ and rotated once per day for 4 days (to reach E4). Embryo removal was performed by windowing eggs and cutting major blood vessels to the embryonic disc tissue. Each extracted embryo was placed in a dish containing PBS, set on a 37°C-heated plate, and decapitated. The conotrunucus and sino venosus were severed to obtain the whole heart tube, which was then incubated at 37°C in pre-warmed chick heart media (α-MEM supplemented with 10% FBS and 1% penicillin/streptomycin) for at least 1 hour prior to use.

*Transfection/knockdown* GFP-lamin-A was a gift from Dr. David Gilbert of Florida State University, Tallahassee, USA [41]; GFP-KU80 was a gift from Dr. Stuart L. Rulten of the University of Sussex, Brighton, UK [42]; and mCherry-cGAS was a gift from Dr. Roger A. Greenberg of the University of Pennsylvania, Philadelphia, USA [43]. We used small interfering RNAs (siRNAs) purchased from Dharmacon (ON-TARGETplusSMARTpool siLMNA, L-004978-00; and non-targeting siRNA, D-001810-10). For cells: we transfected either siLMNA (25 nM) or GFP/mCherry (0.5 ng/mL) with 1 μ g/mL Lipofectamine 2000 (Invitrogen, Life Technologies) for 72 hours (siLMNA) or 24 hours (GFP/mCherry) in corresponding media supplemented with 10% FBS. Knockdown efficiency of siLMNA was determined by immunofluorescence microscopy and by Western blot via standard methods. For embryonic chick hearts: we transfected GFP and mcherry (0.2–0.5 ng/ml) as described above.

*Immunostaining* Cells were fixed in 4% paraformaldehyde (PFA; MilliporeSigma) for 15 minutes, permeabilized by 0.5% Triton-X 100 (MilliporeSigma) for 10 minutes, and blocked with 5% bovine serum albumin (BSA; MilliporeSigma) for 30 minutes. Cells were then incubated in primary antibodies overnight at 4°C. Primary antibodies used were lamin-A/C (1:500, mouse, Cell Signaling #4777S) and lamin-B1 (1:500, rabbit, Abcam ab16048). The next day, cells were incubated in secondary antibodies (1:500, donkey anti-mouse or anti-rabbit, Thermo Fisher) for 90 minutes before DNA was stained using 8 μM Hoechst 33,342 (Thermo Fisher) for 15 minutes. Unless otherwise specified, steps were carried out at room temperature with gentle agitation by orbital shaker. ProLong Gold Antifade (Invitrogen, Life Technologies) was used as a mountant. Embryonic chick hearts were fixed in 4% PFA for 45 minutes, washed using PBS, and permeabilized using 0.5% Triton-X 100 (MilliporeSigma) for 30 minutes. The hearts were incubated in 16 μM Hoechst 33342 (Thermo Fisher) for 2 hours prior to being mounted between two glass coverslips.

*Imaging* Confocal imaging of fixed cells and tissues was done on a Leica TCS SP8 system with 63×/1.4-NA oil-immersion and 40×/1.2-NA water-immersion objectives. Live imaging of cells and tissues was performed under normal culture conditions (37°C and 5% CO_2_, complete medium). Cells were live-imaged using either an EVOS FL Auto Imager (Figure 1b,c) or a Zeiss Axio Observer 7 (Figure 1d), both with 40× objectives; embryonic chick hearts were live-imaged using an Olympus I81 with a 4× objective and a CCD camera. Epifluorescence imaging of fixed progeria cells was obtained using an Olympus IX71 with a digital EMCCD camera equipped with a 40×/0.6-NA objective.

*Image analysis* Image analysis was performed in ImageJ [44].

Measuring lamin-B-to-A ratio: To measure lamin-B-to-A ratio at a nuclear pole, lamin-B and lamin-A intensity profiles were generated along the nucleus’s major axis, originating at the pole of interest (e.g. Figure 1b). The lines used to generate these profiles were ~1.5 µm wide, encompassing multiple pixels, to reduce noise. Each intensity profile was min-max normalized, as follows:

$$\frac{\left(value-\min\right)}{\left(\max-\min\right)}.$$

From these plots, we computed mean lamin-B and mean lamin-A intensity within $a{\;}_{\;}1$ µm distance of the pole (i.e. from *x* = 0 to *x*≈ 1 µm in the plots), and then took the ratio of those two values. Averaging intensities over $a{\;}_{\;}1$ µm distance, rather than taking the intensity at a single point, was intended to reduce noise. We used the same approach to measure lamin-B-to-A ratio at nuclear sides, except intensity profiles were generated along the minor rather than major axis.

Measuring curvature: At the point of interest along the inner nuclear surface, an osculating circle – or ‘circle of best fit’ – was drawn to overlap the nuclear surface for a distance of 2 µm into the cell. Curvature was then calculated as 1/*R*, where *R* is the radius of the osculating circle. Similarly, the curvature of a given pore/pipette was taken to be the inverse of the pore/pipette radius.

Identifying nuclear blebs: To identify nuclear blebs/scars in a given image, we superimposed lamin-A fluorescence signal (green) on top of lamin-B fluorescence signal (red) such that nuclear regions of high-lamin-A/low-lamin-B (i.e. high-green/low-red) were easily detected by eye. For the progeria stem cells, which exhibit very weak staining by lamin-A/C antibodies, local loss of lamin-B was the sole criterion used to identify blebs/scars.

Measuring lamina and DNA dilution in micropipettes: To quantify lamin(-A or -B) or DNA dilution at the leading tip of an aspirated nucleus, we measured the average background-subtracted lamin/DNA intensities of the ‘tip’ and ‘inside’ regions (Figure 4e, inset). Intensities were measured within boxes of area approximating the pipette radius squared. Lamin/DNA dilution was then calculated as the ratio of ‘inside’ intensity to ‘tip’ intensity – minus 1 so that dilution = 0 if the leading tip shows zero lamin loss. ‘Relative LMNB1’ (Figure 4c-ii) was calculated as simply the ratio of ‘tip’ intensity to ‘inside’ intensity.

Measuring DNA dilution in ‘spindle-shaped’ nuclei: DNA dilution in ‘spindle-shaped’ nuclei (Figure 4f, inset) was quantified very similarly to DNA dilution in micropipettes, as described above, except ‘pole’ and ‘center’ regions were used in place of ‘tip’ and ‘inside’ regions; ‘center’ = center of the nucleus. Intensities were measured within boxes of${\;}_{\;}2$ × 2 µm.

*Pore migration*Cells were seeded at a density of 4.5 × 10^5^ cells/cm^2^ on commercially available polycarbonate filter membranes (Corning) with pore diameters of 3, 5, or 8 µm and on etched membranes with modified pore diameters of 2.25, 4, 6, or 7 µm. Pore etching was performed as previously described [21,45]. Complete medium was added to the Top and Bottom of each membrane such that no nutrient gradient was established; for the hypoosmotic stress experiments, complete medium was replaced by a 2:3 mixture of high glucose DMEM and ddH_2_O, supplemented with 10% FBS and 1% penicillin/streptomycin. Cells were allowed to migrate from membrane Top to Bottom over the course of 24 hours under normal culture conditions (37°C and 5% CO_2_). After the 24-hour migration period, each membrane – with un-migrated cells attached on Top and migrated cells attached on Bottom – was fixed, stained, and imaged by confocal microscopy as described above (Immunostaining, Imaging). Membranes were excised from their plastic inserts prior to cell permeabilization.

*Micropipette aspiration*To prepare for aspiration, cells were detached using 0.05% Trypsin-EDTA (Gibco), and incubated in 0.5 µg/mL latrunculin-A (MilliporeSigma) and 8 µM Hoechst 33342 for 30 minutes at 37°C. During aspiration, cells were resuspended in PBS with 1% BSA and 0.2 µg/mL latrunculin-A. Pipette diameters varied from ~2-8 µm, and aspiration pressures varied from ~1 to 10 kPa; see figures and figure legends for the parameters of each experiment. Aspiration was monitored over ~5-60 minutes and imaged using a Nikon TE300 with a 60×/1.25-NA oil-immersion objective and a digital EMCCD camera (Photometrics).

*Drug perturbations*To image cells undergoing mitosis, we synchronized A549 cells at G2- or M-phase by treating with 50 nM nocodazole for 16 hours. Nocodazole was then gently washed out with pre-warmed PBS ×5, allowing cells to proceed through mitosis. To inhibit myosin II, progeria stem cells and embryonic chick hearts were treated with 25 µM blebbistatin for 2 hours prior to fixation and staining. In the pore migration assay with myosin II inhibition, 20 µM blebbistatin was added to the Bottom of the filter membrane for the entire migration period. Adding blebbistatin only to the Bottom – as opposed to both Bottom and Top – has proven sufficient to suppress migration rate as well as nuclear rupture [21], possibly because the cells migrate by extending actin-rich protrusions through the pores that are then followed by myosin-II assemblies [46,47]. In every case, controls were treated with an equal concentration of vehicle solvent DMSO.

*Statistics* All statistical analyses were conducted using Microsoft Excel and Python. Statistical comparisons were made by unpaired two-tailed Student’s *t*-test and were considered significant if *p* < 0.05. Unless mentioned specified, *n* indicates the number of samples, cells, or wells quantified in each experiment.

## Supplementary Material

Supplemental Material

## Data Availability

Data that support the findings of this study are openly available in figshare at https://doi.org/10.6084/m9.figshare.19173497.v1
