# Supplementary figures and images for: Gaussian curvature dilutes the nuclear lamina, favoring nuclear rupture, especially at high strain rate
Source: Nucleus. 2022 Mar 16;13(1):129–43. doi: 10.1080/19491034.2022.2045726 (PMC8928808; doi:10.1080/19491034.2022.2045726)

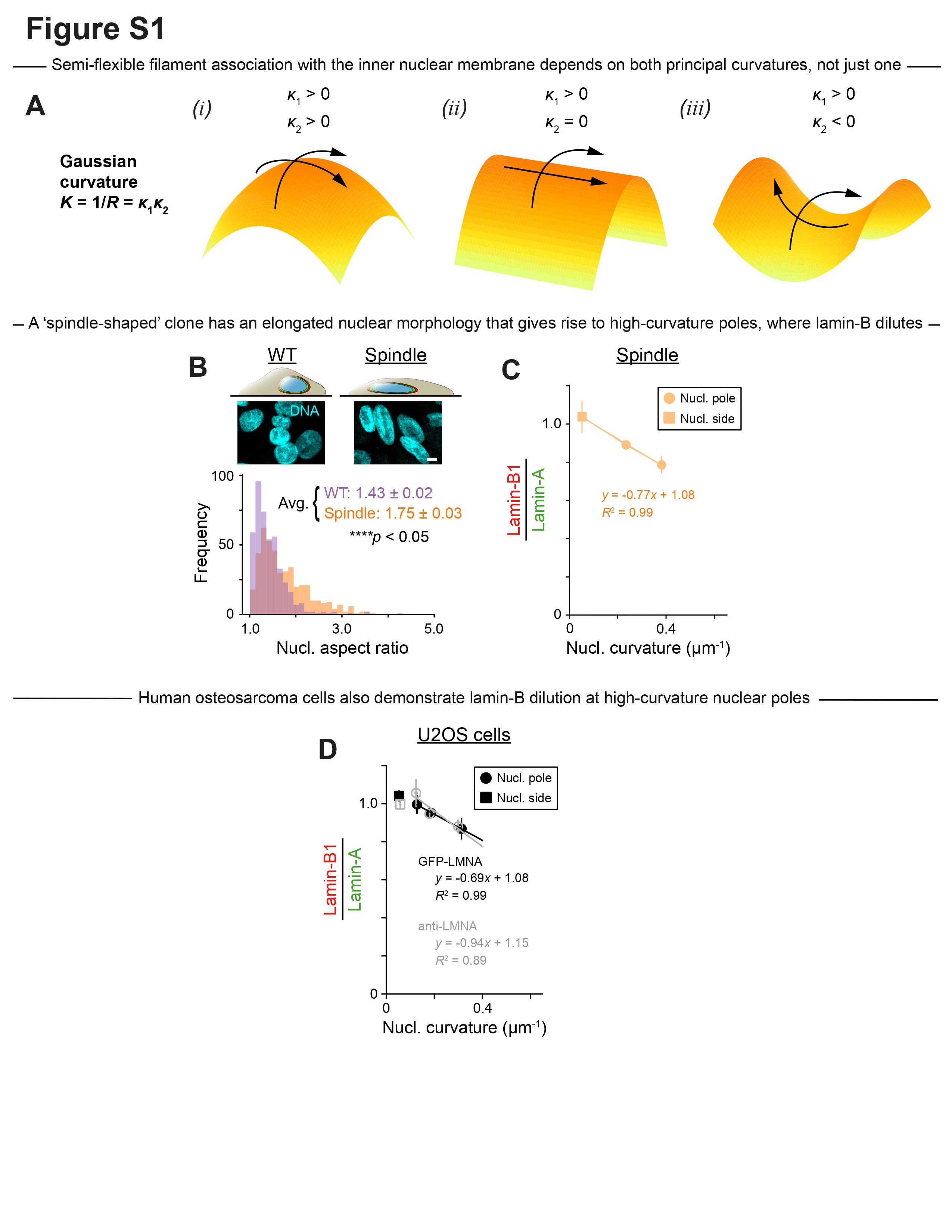

Supplement: Supplemental Material [file KNCL_A_2045726_SM0738.zip › Figure S1.jpg]

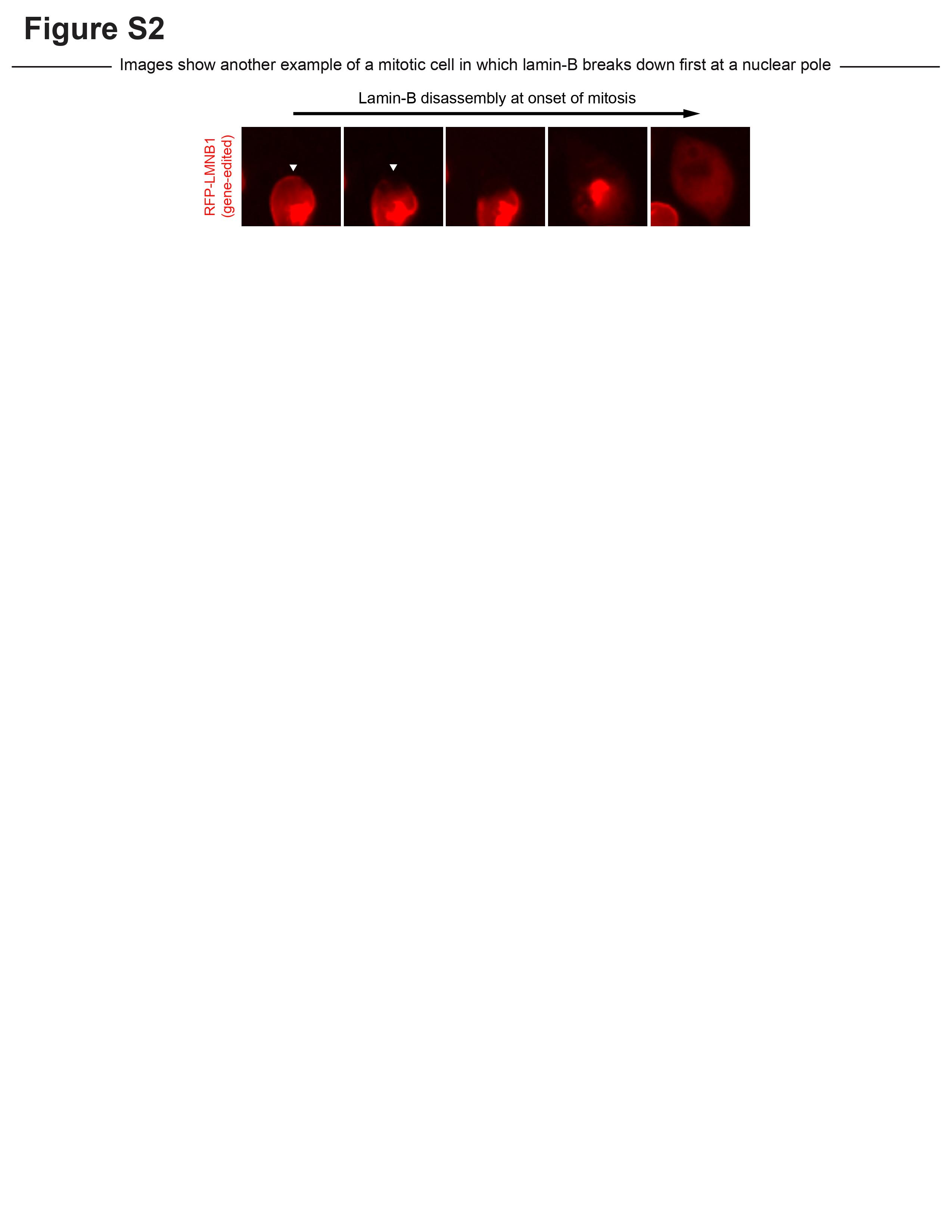

Supplement: Supplemental Material [file KNCL_A_2045726_SM0738.zip › Figure S2.jpg]

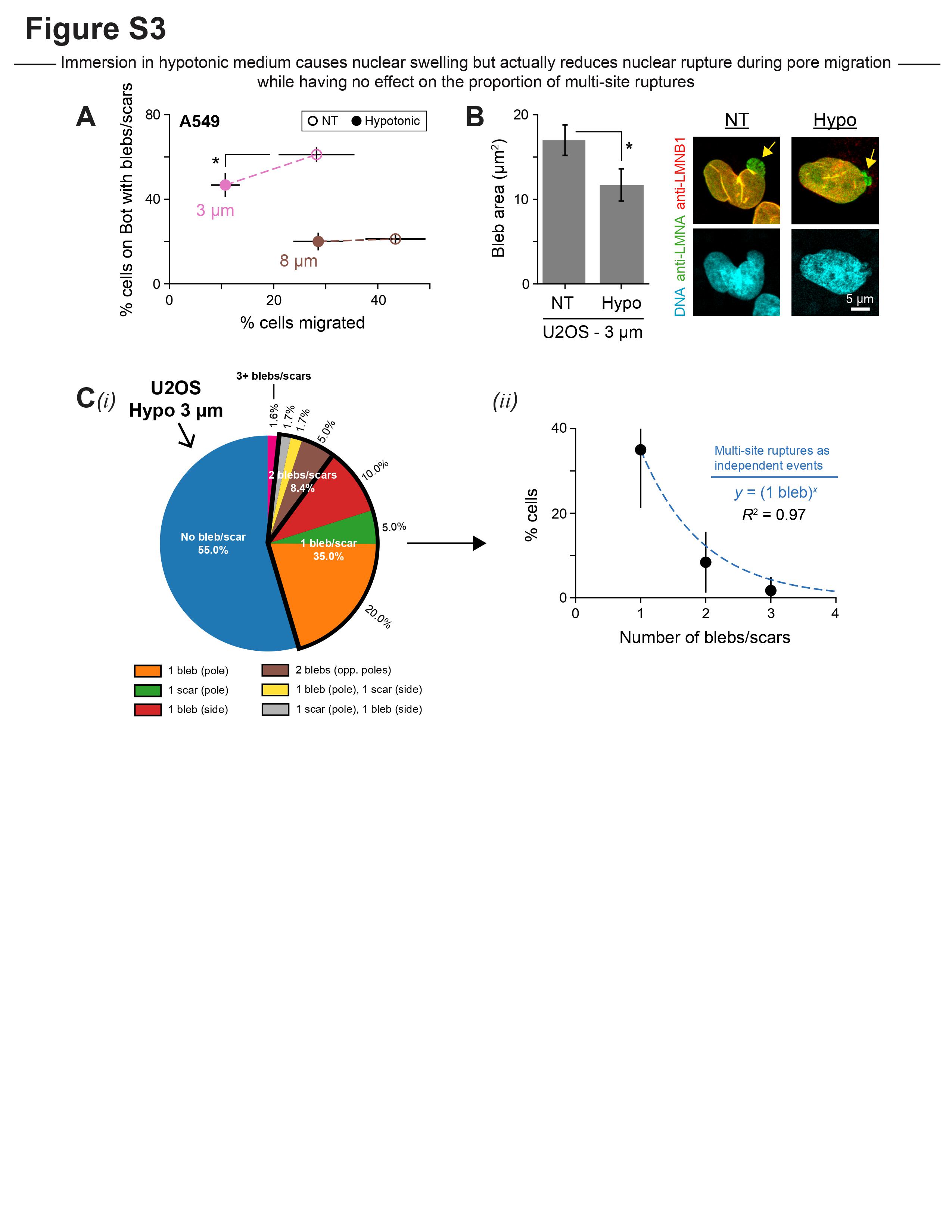

Supplement: Supplemental Material [file KNCL_A_2045726_SM0738.zip › Figure S3.jpg]

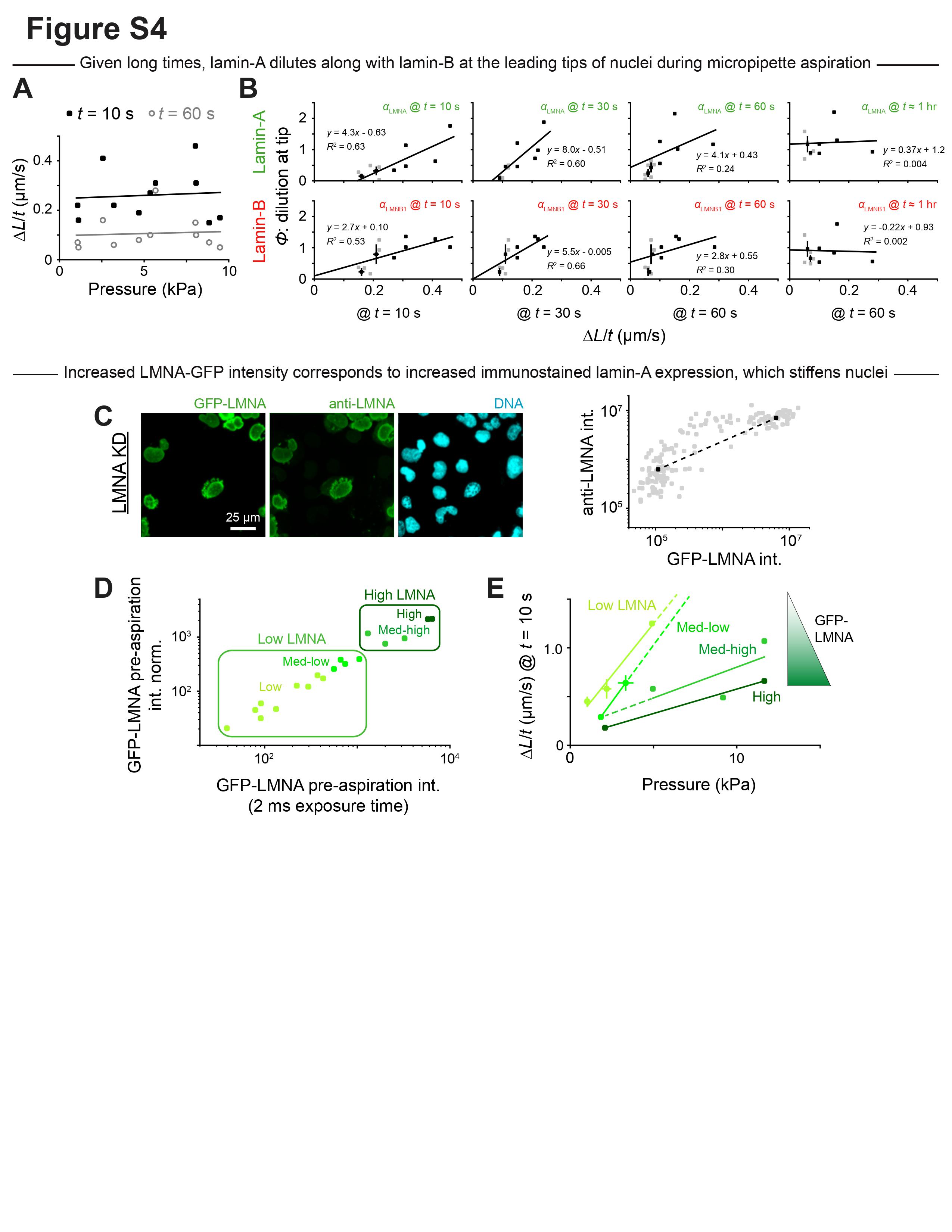

Supplement: Supplemental Material [file KNCL_A_2045726_SM0738.zip › Figure S4.jpg]
